# A Roadmap for Fixing the Heart: RNA Regulatory Networks in Cardiac Disease

**DOI:** 10.1016/j.omtn.2020.04.007

**Published:** 2020-04-25

**Authors:** Rong Tang, Tianxin Long, Kathy O. Lui, Yili Chen, Zhan-Peng Huang

**Affiliations:** 1 Department of Cardiology, Center for Translational Medicine, Institute of Precision Medicine, The First Affiliated Hospital, Sun Yat-sen University, Guangzhou, China; 2 NHC Key Laboratory of Assisted Circulation, Sun Yat-sen University, Guangzhou, China; 3 Department of Chemical Pathology, Li Ka Shing Institute of Health Sciences, The Chinese University of Hong Kong, Prince of Wales Hospital, Shatin, Hong Kong SAR 999077, China

**Keywords:** heart disease, diagnosis and therapy, RNA regulatory network, non-coding RNA, microRNA, micropeptide

## Abstract

With the continuous development of RNA biology and massive genome-wide transcriptome analysis, more and more RNA molecules and their functions have been explored in the last decade. Increasing evidence has demonstrated that RNA-related regulatory networks play an important role in a variety of human diseases, including cardiovascular diseases. In this review, we focus on RNA regulatory networks in heart disease, most of which are devastating conditions with no known cure. We systemically summarize recent discoveries of important new components of RNA regulatory networks, including microRNAs, long non-coding RNAs, and circular RNAs, as well as multiple regulators that affect the activity of these networks in cardiac physiology and pathology. In addition, this review covers emerging micropeptides, which represent short open reading frames (sORFs) in long non-coding RNA transcripts that may modulate cardiac physiology. Based on the current knowledge of RNA regulatory networks, we think that ongoing discoveries will not only provide us a better understanding of the molecular mechanisms that underlie heart disease, but will also identify novel biomarkers and therapeutic targets for the diagnosis and treatment of cardiac disease.

## Graphical Abstract

**Figure:**
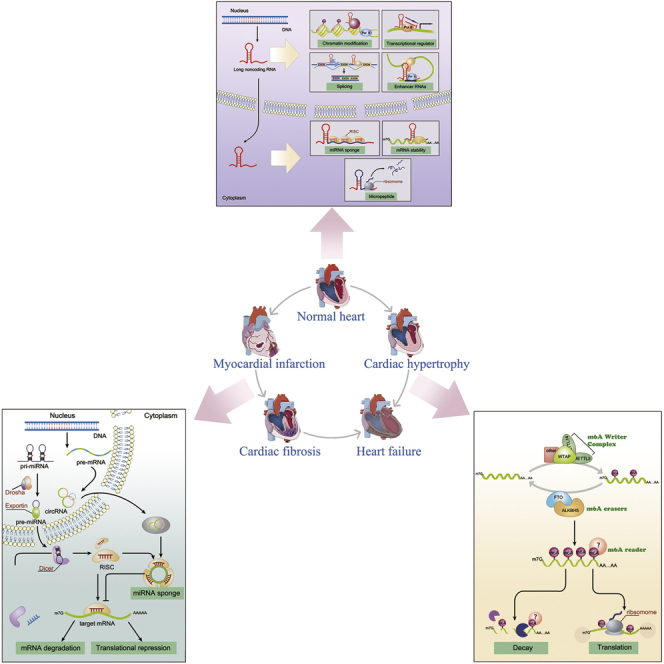

## Teaser

Heart disease is the leading cause of death around the world with no known cure in many cases. Recent discoveries of important new components of RNA regulatory networks in cardiac pathology reviewed herein identify potential novel biomarkers and therapeutic targets for the diagnosis and treatment of this devastating disease.

## Main Text

Cardiac disease is the leading cause of death and disease around the world. In response to external stress or stimulus, the heart maintains homeostasis through dynamic remodeling. In the beginning of remodeling, these adaptations are an attempt to compensate for contractile dysfunction. As cardiac remodeling progresses, compensatory changes are gradually replaced by decompensatory changes. This transition leads to contractile and conduction dysfunction and progression toward heart failure.^1^ When the heart is confronted with serious pathological changes, such as the creation of collagenous, non-contractile scar tissue, thinning of the myocardial wall, or progressive enlargement and dilation of the ventricle, patients have very poor prognosis and an increased risk of death.^2^ Despite significant progress in the treatment of cardiac disease, including heart failure, in the past decade, there remains a lack of therapeutic options that can significantly alter the morbidity and mortality. Consequently, discovery of novel therapeutic targets is urgently required to develop effective treatments for heart disease.

Most research has focused on coding genes involved in the occurrence and progression of cardiac disease. However, the vast majority of the mammalian genome that is transcriptionally active (about 75%–90%) does not encode proteins, as only ∼2% of the DNA encodes proteins.^3^ Therefore, exploration of RNA regulatory networks is imperative, as increasing evidence indicates that non-coding RNAs (ncRNAs) participate in regulating the expression of protein-coding genes. ncRNAs include a variety of functional RNA species, and among all ncRNAs, microRNAs (miRNAs), long ncRNAs (lncRNAs), and circular RNAs (circRNAs) have received the most attention with respect to physiology and pathophysiology, including cardiovascular biology and disease. Recently, it has been discovered that previously “mislabeled” ncRNAs encode stable and functional peptides through short open reading frames (sORFs), and the micropeptides produced from these sORFs participate in the regulation of the physiological function of the heart.^4^ Alternatively, most RNAs undergo a series of modifications after transcription. There are more than 100 known modifications of RNA molecules,^5^ which affect processes such as splicing, nucleation, stabilization, and translation of mRNAs, thereby regulating gene expression. Among these, *N*^6^-methyladenosine (m6A) is one RNA modification that is closely associated with human disease. Although early research on m6A RNA modifications focused on tumor biology, it has recently been shown that m6A modifications are involved in heart disease.^6^^,^^7^

In this review, we summarize the latest research of ncRNA and RNA modifications in heart disease, including cardiac remodeling, fibrosis, and regeneration. We also discuss recent developments and challenges for the development of diagnostic and therapeutic applications of ncRNAs in cardiac disease.

### ncRNAs Play Important Roles in Heart Disease

#### miRNAs Mediate Post-Transcriptional Regulation of Gene Expression in Heart Disease

miRNAs are small, single-stranded ncRNAs with a length of 20–22 nt, which play a crucial role in regulating posttranscriptional gene expression by guiding their target mRNAs to the RNA-induced silencing complex (RISC).^8^ *Lin-4* of *C. elegans* was the first documented miRNA in the early 1990s. This molecule inhibited expression of target genes to regulate developmental timing in worm larvae.^9^ Subsequent studies showed that one-third of the genes in the human genome are regulated by miRNAs,^10^ which indicated that miRNAs play a critical role in various biological processes. Large amounts of data concluded that miRNAs are involved in virtually every cellular process, including proliferation, differentiation, apoptosis, and tumorigenesis.^11^, ^12^, ^13^ Furthermore, accumulating evidence reveals that miRNAs are closely connected to the regulation of cardiac physiology and pathology^14^^,^^15^ (Table 1).

**Table 1:** List of miRNA-Mediated Regulation and Cardiac Function Summarized in This Review

miRNA	Upregulated/Downregulated	Potential Target	Cardiac Function	Refs.
**Hypertrophy and Fibrosis**				

miR-208a	up	THRAP1, myostatin	cardiac hypertrophy	^17^^,^^18^
miR-208a	down	MED13/NcoR1	accelerate the conversion from compensated RVH to decompensated heart failure	^19^
miR-1	down	FBLN2, TWF1, CALM1, CALM2, MEF2A, MYLK3, RasGAP, Cdk9, Rheb	inhibition of cardiac hypertrophy	22, 23, 24, 25, 26, 27, 28, 29, 30
miR-21(CF)	up	SPRY1, Jagged1, mt-Cytb	cardiac fibrosis; myocardial fibroblast proliferation and fibroblast-to-myofibroblast transformation; reduce blood pressure and attenuate cardiac hypertrophy in SHRs	^39^^,^^43^^,^^159^
miR-133	down	CTGF, RHOA, CDC42, NELF-a/WHSC2	inhibition of cardiac hypertrophy	^160^^,^^161^
miR-155	down	Jarid2,	cardiac hypertrophy and cardiac remodeling	^162^
miR-155 (CF)	up	TP53INP1	collagen deposition and fibrosis	^163^
miR-222	up	HMBOX1, p27, HIPK1/2	inhibition of cardiac hypertrophy	^164^
miR-221/222 (CF)	down	JNK1, TGF-β1, TGF-β2, ETS-1	inhibition of fibroblast activation and differentiation	^165^
miR-15 family	up	Sirt4, MO25, SIRT3, TGFβR1, p38, SMAD3, SMAD7	inhibition of hypertrophy	^48^^,^^50^^,^^166^^,^^167^

**Cardiac Ischemic Disease**				

miR-1	up	MYOCD, Bcl2, Hsp90aa1, LXRα	apoptosis	31, 32, 33^,^^168^
miR-208	up	BAX, CHD9, QKI15	apoptosis	169, 170, 171
miR-126	up	ERRFI1	anti-apoptosis	^172^
miR-499	down	CnAα/β, PDCD4, PACS2	anti-apoptosis	173, 174, 175
miR-195	up	CHEK1	inhabitation of proliferation	^52^^,^^176^
miR-15 family	up	SMAD7, Bcl2, β2-AR, c-myb, LC3BII, MFN2, ARL2, MAPK3, CIAPIN1	apoptosis	53, 54, 55, 56, 57, 58, 59, 60, 61, 62

**Cardiac Arrhythmias**				

miR-208a	down	GATA4	cardia conduction defect	^18^
miR-1/133	up	GJA1,KCNJ2, HCN2,HCN4,NCX1,B56α,CACNA1C,IRX5	ventricular arrhythmia; cardiac conduction slow	^34^^,^^36^, ^37^, ^38^^,^^177^
miR-328	up	Cacna1c, Cacnb2	atrial fibrillation	^178^
miR-499	down	KCCN3, CACNB2	atrial fibrillation	^179^^,^^180^

THRAP1, thyroid hormone receptor-associated protein 1; MED13/NcoR1, mediator of transcription 13/nuclear receptor corepressor 1; FBLN2, fibulin-2; TWF1, twinfilin-1; CALM1/2, calmodulin 1/2; MYLK3, myosin light chain kinase 3; RasGAP, Ras GTPase-activating protein; MEF2A, myocyte enhancer factor 2; Cdk9, cyclin-dependent kinase 9; Rheb, Ras homolog enriched in brain; CF, cardiac fibroblast; SPRY1, sprouty homolog 1; mt-Cytb, mtDNA-encoded cytochrome *b*; CTGF, connective tissue growth factor; RHOA, a GDP-GTP exchange protein associated with cardiac growth; CDC42, a signaling kinase involved in pathological hypertrophy; NELF-A/WHSC2, a nuclear factor correlated with cardiogenesis; Jarid2, jumonji, AT-rich interactive domain 2; TP53INP1, tumor protein p53-inducible nuclear protein 1; HMBOX1, homeobox containing 1; HIPK1/2, homeodomain interacting protein kinase 1/2; JNK1, c-Jun N-terminal kinase 1; ETS-1, ETS proto-oncogene 1; SIRT3/4, sirtuin 3/4; MYOCD, myocardin; Bcl-2, B cell CLL/lymphoma 2; BAX, BCL2-associated X; QKI15, RNA-binding protein Quaking 15; CHD9, chromodomain helicase DNA-binding protein 9; ERRFI1, ERBB receptor feedback inhibitor 1; CnAα/β, calcineurin catalytic subunits; PDCD4, programmed cell death 4; PACS2, phosphofurin acidic cluster sorting protein 2; CHEK1, checkpoint kinase 1; β2-AR, β2 adrenergic receptor; MFN2, mitofusin 2; ARL2, ADP-ribosylation factor-like protein 2; MAPK3, mitogen-activated protein kinase 3; CIAPIN1, cytokine-induced apoptosis inhibitor 1; GJA1, gap junction protein α1; KCNJ2, potassium inwardly rectifying channel subfamily J member 2; HCN2/HCN4, hyperpolarization activated cyclic nucleotide gated potassium and sodium channel 2/4; NCX1, sodium/calcium exchanger protein; CACNA1C, calcium voltage-gated channel subunit α1C; IRX5, iroquois homeobox 5; CACNB2, voltage-dependent calcium channel β2 subunit; Cacna1c, calcium voltage-gated channel subunit 1C; Cacnb2, calcium voltage-gated channel auxiliary subunit 2; KCNN3, potassium calcium-activated channel subfamily N member 3.

*Cardiac-Enriched miRNAs.* A subset of miRNAs are enriched in the heart, such as miR-1, miR-133, miR-208, and miR-499.^16^ miR-208 was one of the first miRNAs reported to be involved in cardiac hypertrophy.^17^ Both gain- and loss-of function studies demonstrated that miR-208 was required for cardiac hypertrophy by targeting the thyroid hormone receptor-associated protein 1 (THRAP1). miR-208a, which is encoded within an intron of *Myh6*, and miR-208b, which is encoded within an intron of *Myh7*, are members of a miRNA family that is differentially expressed during cardiac development and pathology. Callis et al.^18^ demonstrated that overexpression of miR-208a is sufficient to induce cardiac hypertrophy, accompanied with increased β-myosin heavy chain (β-MHC) expression. miR-208a targets Thrap1 and myostatin, two important negative regulators of hypertrophic growth. In addition, miR-208a is required for normal cardiac conduction. Electrocardiograms (ECGs) showed that approximately 80% of *Mir208a*^*–/–*^ mice lacked P waves and had prolonged PR intervals compared to wild-type mice. An additional study confirmed that miR-208a regulates expression of Cx40 and Hop through the transcriptional cofactor GATA4. Furthermore, a recent study reported that miR-208 is progressively downregulated as right ventricular hypertrophy progressed because of pulmonary hypertension. miR-208 also inhibited the expression of Mef2 through the Med13-NCoR1 axis, and therefore suppresses the disease transition from compensation to decompensation.^19^

miR-1 is another well-studied, cardiac-enriched miRNA. miR-1-1 and miR-1-2 are members of the miR-1 family and are located at separate chromosomal loci. miR-1 and miR-133a form a miRNA gene cluster and are co-expressed during cardiomyocyte differentiation and proliferation.^20^ Sayed et al.^21^ showed that several targets of miR-1 are involved in progressive myocardial hypertrophy and cardiac remodeling, including Ras GTPase-activating protein (RasGAP) and cyclin-dependent kinase 9 (Cdk9), activators of cardiac hypertrophy,^22^^,^^23^ Ras homolog enriched in brain (Rheb), an upstream activator of protein synthesis, and the cell growth-related mammalian target of rapamycin (mTOR)/S6 kinase pathway.^24^^,^^25^ Recent studies confirmed that miR-1 suppresses cardiac hypertrophy by inhibiting the expression of various downstream targets, including fibulin-2 (FBLN2),^26^ twinfilin-1 (TWF1),^27^ CALM1 and CALM2, MEF2A,^28^ MYLK3,^29^ and GATA4.^30^ In addition, the serum level of miR-1 and miR-133 is elevated in animal models and human patients with acute myocardial infarction (AMI). Inhibition of miR-1 with antisense oligonucleotides attenuates myocardial apoptosis by targeting Bcl2.^31^ Other studies reveal that miR-1 also represses expression of Hsp90aa1 and the liver X receptor α (LXRα), which affects cardiomyocyte apoptosis during myocardial infraction (MI).^32^^,^^33^ Similar to miR-208a, miR-1 is also required for normal cardiac electrophysiology. Widening of the QRS complex and a prolonged QT interval were observed in miR-1-transfected hearts.^34^ miR-1 repressed expression of its targets, GJA1 and KCNJ2, and led to a lower protein level of Cx43 and Kir2.1, resulting in a propensity for arrhythmia. In addition, it has been reported that miR-1 and miR-133 targeted several ion channel- and gap junction-associated genes, such as HCN2, HCN4,^35^ NCX1,^36^, B56α,^37^ CACNA1C, and IRX5.^38^

Therefore, these cardiac-enriched miRNAs seem to be housekeepers of cardiomyocytes. They maintain cardiomyocyte physiology, including assembly and function of the contractile apparatus as well as controlling electrophysiological function, to ensure efficient and coordinated pumping of blood to the circulation.

*Ubiquitously Expressed miRNAs.* Other than cardiac-enriched miRNAs, some ubiquitously expressed miRNAs also play important roles in cardiac pathology. Previous studies have shown that miR-21 is closely involved in the pathological progression of multiple cardiac abnormalities, including aberrant remodeling, arrhythmia, heart failure, and infarction. Thum et al.^39^ found that miR-21 activated the ERK/MAPK (extracellular signal-regulated kinase/mitogen-activated protein kinase) signaling pathway by inhibiting Spry1 expression, thereby promoting cardiac fibroblast activation and growth factor secretion. Interestingly, intravenous injection of antagomiR-21 suppresses myocardial fibrosis and preserves cardiac function; however, the precise mechanism remains poorly understood. It was suggested that fibroblast exosomal-derived miR-21_3p (miR-21∗) is a potent paracrine-acting RNA molecule that induces cardiomyocyte hypertrophy.^40^ A recent study showed that miR-21 plays a key role in myocardial fibroblast activation and myocardial fibrosis following MI by targeting the transforming growth factor β (TGF-β)1/Smad7 signaling pathway.^41^ Interestingly, phosphorylated Smad2 and Smad3, which are downstream effectors of TGF-β signaling, interact with DROSHA to promote processing of primary miR-21 under pressure overload through a feedback loop.^42^ Zhou et al.^43^ also showed that miR-21 promotes myocardial fibroblast proliferation and fibroblast-to-myofibroblast transformation by targeting Jagged1. Also note that loss of miR-21 through genetic engineering could not recapitulate the cardiac phenotype observed as a consequence of antagomiR interference,^44^ indicating that the transient interference with the function of miR-21 could be compensated for by other mechanisms in the long term.

miR-21 also participates in the regulation of cardiomyocyte apoptosis in ischemic cardiomyopathy. It was reported that miR-21 is downregulated in the infarcted region 6 h after AMI.^45^ Additional studies demonstrated that miR-21 inhibits hypoxia-induced apoptosis through the PDCD4/AP-1 (activator protein 1) pathway by targeting PDCD4. Therefore, miR-21 appears to play a protective role in reducing oxidative stress in cardiomyocytes due to ischemia/reperfusion (I/R) injury.^46^^,^^47^

The miR-15 family consists of six members, which possess a common seed sequence, including miR-15a, miR-15b, miR-16-1, miR-16-2, miR-195, and miR-497. Recently, several studies have shown that the miR-15 family plays crucial roles in the pathogenesis of cardiac disease. Tijsen et al.^48^ found that the miR-15 family was upregulated in the hypertrophic heart. Inhibition of miR-15b with locked nucleic acid (LNA)-based antimiRs leads to a significant increase in heart weight, excessive fibrosis, and collagen deposition during hypertrophy. The miR-15 family inhibits canonical and non-canonical TGF-β signaling, which constitutes a critical pathway for cardiac fibrosis and hypertrophy, by targeting multiple direct and indirect genes, including TGFβR1, P38, SMAD3, SMAD7, and endoglin. A previous study indicated that miR-195 plays an essential role in hypertrophic growth and chamber remodeling of the heart in response to pathological signaling.^49^ It was further demonstrated that the elevated expression of miR-195 in hypertrophic cardiomyocytes impedes the formation of LKB1/STRAD/MO25 complexes and activates the AMPK (AMP-activated protein kinase) pathway by suppressing MO25.^50^

Most mammalian cardiomyocytes lose the ability to regenerate shortly after birth. Once the heart is severely damaged by injuries such as those caused by MI, cardiomyocyte replenishment is insufficient to repair the damage.^51^ Porrello et al.^52^ found that multiple miR-15 family members, including miR-195, miR-497, miR-15a, and miR-16, are upregulated in the mouse ventricles between postnatal day 7 and 14. Inhibition of the miR-15 family prevents cardiomyocyte mitotic arrest and improves cardiac function after MI. Additional experiments showed that miR-195 regulates a number of mitotic genes *in vivo* by targeting Chek1. Other studies revealed that the miR-15 family not only regulates cardiomyocyte proliferation and cardiac regeneration, but it also modulates cardiomyocyte apoptosis. Loss of miR-15 family members *in vitro* or *in vivo* renders cardiomyocytes resistant to hypoxia-induced cell death, reduces infarct size, and suppresses cardiac remodeling.^53^ Recent studies further demonstrated that the miR-15 family targets other downstream genes involved in regulating cardiomyocyte apoptosis, such as SMAD7,^54^ Bcl2,^55^, ^56^, ^57^ β2 adrenergic receptor (β2-AR),^58^ c-myb,^59^ LC3B-II,^57^ mitofusin 2 (MFN2),^60^ ADP-ribosylation factor-like protein 2 (ARL2),^53^ MAPK3,^61^ and cytokine-induced apoptosis inhibitor 1 (CIAPIN1).^62^

In summary, a large effort has been expended on investigating these “tiny” miRNAs. Many of the miRNAs listed in Table 1, but not discussed here in detail, participate in the regulation of many aspects of cardiac physiology and pathology.

### lncRNAs Have a Variety of Molecular Functions in Regulating Heart Disease

New technologies for genome-wide, massively parallel sequencing have led to the discovery that vast regions of the mammalian genome are actively transcribed into RNA. Surprisingly, all protein-coding sequences originate from about only 1.5% of the human genome sequence.^63^ As a result, numerous non-coding transcripts have been identified. lncRNAs belong to a class of ncRNAs with a length of more than 200 nt. Because of a huge effort, more and more lncRNAs are now known to have significant regulatory functions in cardiovascular biology.^64^^,^^65^ Herein, we have cataloged lncRNAs with important functions in cardiac remodeling, including those involved in hypertrophy, apoptosis, necrosis, and fibrosis^66^^,^^67^ (Table 2).

**Table 2:** List of Cardiac Function of lncRNAs and Their Molecular Mechanisms Summarized in This Review

lncRNA	Upregulated/Downregulated	Potential Mechanism	Effect	Refs.
**Hypertrophy**				

Mhrt	down	interacts with Brg1	inhibits developing heart failure	^68^^,^^69^^,^^181^
Chaer	Up	interacts with PRC2	promotes cardiac hypertrophy	^70^^,^^182^
Chrf	Up	sponge for miR-489	promotes cardiac hypertrophy	^71^

**Apoptosis and Autophagy**				

APF	Up	sponge for miR-188-3p	promotes deregulated autophagy and cell death	^76^
CAIF	down	interacts with p53	inhibits autophagy	^77^
MALAT1	Up	sponge for miR-203	worsens cardiomyocyte inflammation and apoptosis	^78^

**Electrical Activity**				

MALAT1	up	sponge for miR-200c	regulates transient outward potassium current	^79^

**Cardiac Fibrosis**				

MALAT1	Up	sponge for miR-145	promotes cardiac fibrosis and deteriorates cardiac function after MI	^80^
Wisper	Up	interacts with TIAL1	promotes cardiac fibrosis	^81^
MEG3	down	interacts with p53	promotes cardiac fibrosis and impaired diastolic performance	^82^
GAS5	down	sponge for miR-21	inhibits cardiac fibrosis	^83^

Mhrt, Myheart; Brg1, also known as Smarca4 (SWI/SNF-related, matrix-associated, actin-dependent regulator of chromatin, subfamily a, member 4); Chaer, cardiac hypertrophy-associated epigenetic regulator; PRC2, polycomb repressive complex 2; Chrf, cardiac hypertrophy-related factor; APF, autophagy-promoting factor; CAIF, cardiac autophagy inhibitory factor; MALAT1, metastasis-associated lung adenocarcinoma transcript1; MI, myocardial infarction; Wisper, Wisp2 super-enhancer-associated RNA; TIAL1, TIA1 cytotoxic granule-associated RNA-binding protein-like 1; MEG3, maternally expressed gene 3; GAS5, growth arrest-specific 5.

Cardiac hypertrophy is an adaptive response by the heart to counteract cardiac overload to maintain output. However, sustained hypertrophy often leads to heart failure. Recently, lncRNA Myheart (Mhrt), which originates the from MYH7 locus and is enriched in adult hearts, was found to protect the adult heart from pathological hypertrophy by interacting with the helicase domain of Brg1 and inhibiting the function of Brg1, a chromatin-remodeling factor that is activated by stress and triggers aberrant gene expression and cardiomyopathy.^68^^,^^69^ Conversely, lncRNA Chaer (cardiac hypertrophy-associated epigenetic regulator) is required for the pathogenesis of cardiac hypertrophy. Chaer interacts with PRC2 and interferes with the targeting of the PRC2 complex to genomic loci, which inhibits PRC2-dependent histone H3 lysine 27 trimethylation at the promotor of prohypertrophic genes and the activation of their expression.^70^ The molecular mechanisms that underlie lncRNA regulation of cardiac hypertrophy are not limited to their action as a decoy for epigenetic regulators, as they also function as endogenous sponges for miRNAs. For example, lncRNA Chrf serves as a competing RNA by sequestering miR-489 and de-repressing the miR’s target, MYD88.^71^ Furthermore, ROR,^72^ H19,^73^ Plscr4,^74^ and MI-associated transcript (MIAT)^75^ regulate cardiac hypertrophy through a similar mechanism by inhibiting the function of different miRNAs.

lncRNAs, such as APF, CAIF, and MALAT1 (metastasis-associated lung adenocarcinoma transcript 1) were reported to regulate cardiomyocyte apoptosis and autophagy in heart disease. Under pathological conditions, upregulating the autophagy promoting factor (APF) de-represses the autophagy gene ATG7 by sequestering miR-188-3p. This leads to abnormal autophagy as well as cell death.^76^ In contrast, lncRNA CAIF (cardiac autophagy inhibitory factor) acts as a cardioprotective factor. CAIF inhibits p53-induced transcription of myocardin by directly binding to its promoter, which leads to the suppression of cardiac autophagy and protection of the heart during MI.^77^ During I/R injury, MALAT1 is highly expressed in heart, and it leads to a more severe cardiomyocyte inflammation and apoptosis by sequestering miR-203.^78^

Other than cardiac hypertrophy and apoptosis, lncRNAs have also been reported to regulate arrhythmia and fibrosis in heart disease. MALAT1 was reported to regulate electrical activity in an arrhythmic rat model by modulating expression of the miR-200c-HMGB1 axis in cardiomyocytes. Expression of transient outward potassium current and Kv4.2/Kv4.3 channel proteins are regulated via HMGB1 when MALAT1 is knocked down.^79^ In addition, knockdown of MALAT1 inhibits AngII-induced fibroblast proliferation and collagen synthesis, and then suppresses cardiac fibrosis following MI by suppressing TGF-β1 activity via miR-145.^80^ Other lncRNAs, such as Wisp2 super-enhancer-associated RNA (Wisper),^81^ MEG3,^82^ and GAS5,^83^ have also been reported to participate in the regulation of cardiac fibrosis through various molecular mechanisms.

As mentioned above, a large number of lncRNAs play important roles in cardiac remodeling during stress. It is also noteworthy that several lncRNAs, including Braveheart (Bvht)^84^ and Fendrr,^85^ are critical to cardiac lineage commitment and lead to developmental defects in the heart when these lncRNAs are deleted. Both of these lncRNAs interact with the PRC2 complex and epigenetically regulate the cardiac transcriptome during cardiac development.

### circRNAs Primarily Function as a miRNA Sponge in Heart Disease

circRNAs are a class of ncRNA molecules shaped by a covalently closed continuous loop. Previous studies indicated that circRNAs play vital roles in the regulation of gene expression, including miRNA sponge effects, transcriptional and post-transcriptional gene expression regulation, alternative splicing, and protein coding and protein decoy activity. Some of these molecules are expressed in a tissue-specific manner.^86^, ^87^, ^88^, ^89^, ^90^ Recently, it has been shown that circRNAs are closely related to the pathological and physiological processes of various cardiac diseases, such as myocardial ischemia, myocardial fibrosis, cardiac hypertrophy, and heart failure.

An early study on the role of circRNAs in hypertrophy and heart failure revealed decreased expression of circRNA HRCR, which functions as a sponge to sequester cardiac miR-223, in the failing heart. *In vivo* overexpression of HRCR results in increased expression of miR-223’s downstream target ACR,^91^ which is an apoptosis repressor with a CARD domain. ACR plays a crucial role in cardiomyocyte hypertrophy and apoptosis^92^ and protects the heart from hypertrophy and failure. Another interesting study reported that expression of circRNA Foxo3 is significantly higher in aged hearts compared to young hearts. It induces cellular senescence and doxorubicin-induced heart failure by interacting with the anti-senescence proteins ID1 and E2F1, and the anti-stress proteins FAK and HIF-1α. These interactions block the nuclear translocation of these proteins and inhibit their function as transcription factors.^93^

Multiple circRNAs have been reported to regulate apoptosis and survival in heart disease. circRNA cerebellar degeneration-related protein 1 transcript (Cdr1) contains complementary binding sites for miR-7a that may function as miRNA sponges. This circRNA de-represses targets of miR-7a, PARP, and SP1, and it participates in the regulation of apoptosis after MI injury.^94^ In another study, it was shown that mitochondrial fission and apoptosis-related circRNA (MFCAR) plays an essential role in modulating mitochondrial fission and apoptosis by acting as a sponge for miR-652-3p. MFCAR prevents miR-652-3p from binding with mitochondrial membrane-associated protein 18 (MTP18). Knockdown of MFCAR decreases expression of MTP18 and attenuates mitochondrial fission and cardiomyocyte apoptosis in MI injury.^95^ Other than acting as miRNA sponges, circRNAs can interact with proteins and regulate their activities. A recent study reported that circ-Amotl1 binds to AKT and PDK1 and induces their nuclear translocation.^96^ *In vivo*, circ-Amotl1 overexpression enhances cardiomyocyte survival and, therefore, protects the heart in doxorubicin-induced cardiomyopathy.^96^ Furthermore, Zhou et al.^97^ report that a circRNA, autophagy-related circRNA (ACR), protects the heart from I/R injury and reduces the extent of the infarct. Mechanistically, ACR directly binds to Dnmt3B and blocks Dnmt3B-mediated DNA methylation of the promoter of Pink1, which suppresses autophagy via phosphorylating its downstream target, FAM65B.

Emerging evidence indicates that circRNAs also participate in the regulation of cardiac regeneration. Super-enhancer-associated circRNA circNfix was found to enhance expression in the adult heart.^98^ This study showed that circNfix regulates cardiomyocyte proliferation through diverse molecular mechanisms. circNfix functioned as a miRNA sponge to modulate Gsk3β signaling activity by sequestering miR-214. Alternatively, circNfix interacts with Ybx1 (Y-box-binding protein 1) and Nedd4l (an E3 ubiquitin ligase) and enhances the interaction of these two proteins, which induces Ybx1 degradation through ubiquitination. Knockdown of circNfix promotes cardiomyocyte proliferation and angiogenesis and, therefore, attenuates cardiac dysfunction and protects the heart after MI.

Aside from their function for RNA transcripts, a recent study showed that ribosome-associated cardiac circRNAs produce detectable peptides.^4^^,^^99^ The roles of these peptides in cardiac disease are currently unknown and, consequently, provide a new direction for future exploration.

### Micropeptides Encoded by “Non-coding” RNAs in Heart Disease

Micropeptides are a group of protein molecules less than 100–150 aa in length.^100^ Micropeptides are significantly different from bioactive peptides, because the former originate from sORFs, which nest in transcripts identified as lncRNAs and TUFs (transcripts of unknown function), whereas the latter are derived from larger precursor proteins and contain N-terminal signal sequences.^101^ Because they are short, traditional computational prediction programs of protein-coding ORFs excluded these sORFs as false positives.^102^^,^^103^ Studies have shown that some of these sORF have non-classical start codons as well as low sequence conservation, which posed a challenge to uncover these micropeptides in the mammalian genome.^100^

Using emerging technologies and experimental approaches, researchers have begun to address this challenge. For example, Anderson et al.^104^ reported a group of micropeptides, named myoregulin (MLN), phospholamban (PLN), and sarcolipin (SLN). These peptides have similar conserved regions in their peptide sequence as well as a homologous function to inhibit SERCA activity by regulating cardiac calcium uptake in muscle. Another two micropeptides that have functions similar to MLN/PLN/SLN were subsequently identified and named endoregulin (ELN) and another-regulin (ALN).^105^ The search for SERCA-associated regulatory micropeptides did not end there, as the identification of the micropeptide dwarf ORFs (DWORFs) revealed enhanced SERCA activity by displacing the SERCA inhibitors PLN, SLN, and MLN in the mouse heart. So far, DWORF is the only endogenous peptide known to activate the SERCA pump by a physical interaction, resulting in enhanced muscle contraction.^106^

As more micropeptides are identified, the questions of how many micropeptides are present in the heart and whether they share any common features will eventually be answered. For instance, a genome-wide study recently identified micropeptides in diseased hearts. As a result, hundreds of micropeptides were found in human, mouse, and rat hearts. Interestingly, the overall coding sequence for these micropeptides were less conserved than that observed in traditional proteins. Furthermore, this study indicated that many microproteins are produced from sORFs located in lncRNAs identified with previously described cardiac functions, such as Myheart,^69^ chaer,^70^ UPPERHAND (also known as UPH or HAND2-AS1),^107^ ZFAS1,^108^ and TRDN-AS (also known as RP11-532N4.2).^109^ Although the subcellular location of micropeptides varied, most of these localized to mitochondria,^4^ which suggests that micropeptides could have important regulatory functions for mitochondrial biogenesis and function. Indeed, recent studies have shown a micropeptide named MOXI (micropeptide regulator of β-oxidation)^110^ or Mtln (mitoregulin)^111^ interacts with the mitochondrial trifunctional protein (MTP) and several mitochondrial complexes to regulate mitochondrial function, including fatty acid β-oxidation, respiratory (super)complex formation and activity, Ca^2+^ retention, and reactive oxygen species formation.

It is important to recognize that as more micropeptides are identified, it will be necessary to take this into account in future investigations of RNA regulatory networks, especially those determining the function of lncRNAs. To accurately define the function of a “non-coding” gene, the coding potential of a transcript needs to be carefully excluded when investigating the function of lncRNAs. For example, recent studies identified UPPERHAND as a critical lncRNA during cardiac development.^107^^,^^112^ However, a potential coding sORF was also identified in both human and murine UPPERHAND.^4^ Therefore, further studies need to be carried out to determine whether the discovered function of UPPERHAND was derived from the RNA transcript or the micropeptide. Alternatively, more effort should be expended on genome-wide discovery of sORFs to define the noncoding gene. Various approaches, such as computational analyses, ribosome profiling (Ribo-seq [ribosome sequencing]), mass spectrometry, and combinations of these procedures are recommended for accurately identifying protein-coding sORFs.

### Dysregulation of RNA Modifications Is Associated with Heart Disease

RNA molecules often undergo various modifications post-transcriptionally. m6A methylation is one of the most widespread, internal, post-transcriptional modifications of eukaryotic mRNAs, involving the regulation of physiological and pathological activities by modifying mRNA or ncRNA.^113^ Although m6A was first discovered in 1974,^114^ its location in mRNAs and functions are not fully understood. Recently, the dynamics and function of m6A modifications of mRNAs in different biological processes have been intensively investigated. The m6A modifications were recently reported to facilitate cap-independent mRNA translation.^115^ The modification of m6A can be dynamically deposited, removed, and identified by a series of methyltransferases (METTL3/14, WTAP, RBM15/15B, ZC3H13, KIAA1429, and METTL16, termed “writers”), demethylases (FTO and ALKBH5, termed “erasers”), and m6A-binding proteins (YTHDF1/2/3, IGF2BP1 and HNRNPA2B1, termed “readers”).^116^^,^^117^ More and more studies have demonstrated that the abnormal dynamics of methylation of RNA on *N*^6^-adenosines are closely related to tumorigenesis.^118^

Until recently, the connection between m6A RNA modifications and heart disease was yet to be explored. Dorn et al.^6^ demonstrated that m6A modification of a subset of mRNAs was significantly increased in response to a hypertrophic stimulus in cardiomyocytes. As an important enzyme for *N*^6^-adenosine methylation, overexpression of METTL3 was sufficient to induce adaptive cardiac hypertrophy in the heart. Conversely, inhibition of METTL3 expression suppressed the hypertrophic growth of cardiomyocytes. Furthermore, the METTL3 knockout mouse showed gradual pathological changes during aging and stress. Interestingly, m6A modifications were found to occur specifically at MAPK mRNAs, which are important for hypertrophic growth of cardiomyocytes. In an ischemic heart mouse model, Song et al.^119^ have shown that increased Mettl3 activity promoted the association of HNRNPD with Tfeb pre-mRNA by regulating m6A modifications in the Tfeb 3′ UTR, and then decreased Tfeb mRNA stability in hypoxia/reoxygenation-treated cardiomyocytes, which inhibited the autophagic flux and promoted apoptosis of cardiomyocytes. In another recent study, decreased FTO expression and increased m6A RNA modifications were found in failing mammalian hearts and hypoxic cardiomyocytes. Importantly, myocardial overexpression of FTO showed a protective effect in ischemic hearts.^7^ It was shown that loss of FTO leads to abnormal calcium homeostasis and sarcomeric dynamics. In contrast, FTO overexpression selectively increases demethylation of contractile protein-related mRNAs, thereby inducing their expression. In addition, decreased cardiac fibrosis and enhanced angiogenesis were observed in the FTO-overexpressing ischemic myocardium through an unknown mechanism. Future studies may uncover the underlying mechanisms, which could lead to an identification of novel therapeutic strategy for MI. So far, while the m6A modification of mRNA was linked to heart disease, it will be interesting to find out whether m6A modifications of ncRNAs are involved in the pathogenesis of cardiac disease.

Increased mRNA translation is an essential step for cardiac remodeling, in which several key signaling pathways are involved, including AKT^120^ and AMPK.^121^^,^^122^ Similar to m6A modifications, which affect the translational activity of mRNA, the length of the poly(A) tail of the PABPC1 mRNA, coding a poly(A)-binding protein known to promote translation, was reported to be a key modification regulating the translation efficiency of its own mRNA.^123^ Pabpc1 poly(A) tail length was found to be significantly shorter in the adult heart compared to its length in the embryonic heart. This effect is correlated with the translational silencing of Pabpc1 in the adult heart under physiological conditions. The shortening of the poly(A) tail was reversed in the hypertrophic heart. It significantly enhanced the translation of Pabpc1 and triggered the subsequent global mRNA translational enhancement observed in cardiac hypertrophy. Unfortunately, the detailed mechanism of how this modification is regulated remains to be thoroughly explored.

### RNA Molecules Are Potential Targets for Clinical Diagnosis and Gene Therapy for Cardiac Disease

One of the ultimate goals for investigating RNA regulatory networks in cardiac disease is to develop clinical applications with those RNA molecules, which can serve as biomarkers for disease diagnosis/prognosis and/or therapeutic targets.

Other than behaving as regulatory factors in the pathogenesis of cardiac disease, ncRNAs also function as paracrine factors by interacting with proteins to form RNA-protein complexes, as well as with lipids or high-density lipoproteins in the circulation.^124^ These complexes are stable and resistant to RNase degradation. Therefore, some ncRNAs with different expression levels in the serum of healthy and diseased people have the potential to act as biomarkers for the diagnosis of heart disease.

Effective biomarkers are important for assessing post-infarction risk and treatment responses in AMI. miR-1, miR-126, and cTnT expression levels in plasma from patients with AMI are significantly elevated, suggesting that miR-1 and miR-126 could be valuable indicators for AMI.^125^ miR-499 is specifically expressed in cardiomyocytes and only increases after AMI.^126^ Therefore, miR-499 could be an important biomarker for MI, especially NSTEMI.^127^ Other miRNAs, such as miR-208,^128^ miR-133,^129^ miR-1254,^130^ miR-99a,^131^ miR-122-5p,^132^ miR-874-3p,^133^ miR-19b, miR-223, and miR-483-5p,^134^ also have the potential to predict MI as biomarkers. The potential for lncRNAs to serve as biomarkers of cardiac disease has also been investigated. Vausort et al.^135^ demonstrated that levels of circulating lncRNAs aHIF, KCNQ1OT1, and MALAT1 were higher in patients with MI than in healthy volunteers, while levels of the circulating lncRNA ANRIL were lower. A recent study showed that high plasma ANRIL levels were correlated with a high risk of in-stent restenosis (ISR).^136^ Other evidence suggests that HOTAIR,^137^ UCA1,^138^ MHRT,^139^ MIAT,^140^ LIPCAR,^141^ CDR1AS, and ZFAS1^142^ could serve as potential markers for diagnosis and prognosis of AMI or CAD. circRNAs were found to be abundant in circulating blood and more stable than linear RNAs because of the closed-loop structure.^143^ These attributes allow the detection of these circRNAs using a convenient method.^144^ circRNA MICA was found to be downregulated in peripheral blood samples from MI patients.^145^ A study of 472 patients with AMI showed that circRNA MICRA improved the predictive value of a multivariable clinical model and it also improved the risk classification of patients after MI.^146^

The potential of ncRNAs as biomarkers for heart failure was also investigated. Similar to established diagnostic protein biomarkers, such as cTnI, circulating cardiac-enriched miRNAs (myomirs) increased up to 140-fold in advanced heart failure.^147^ In a study of chronic heart failure, miR-660-3p, miR-665, miR-1285-3p, and miR-4491, which were derived from cardiac fibroblasts, were significantly increased in heart and plasma.^148^ Recent studies also showed that many circulating miRNAs were differentially expressed in heart failure, including miR-18a-5p, miR-26b-5p, miR-27a-3p, miR-199a-3p,^149^ miR-499,^150^ miR-155-5p, and miR-595.^151^ Some of these were also demonstrated to be effective in the assessment of risk. For example, a decrease in plasma miR-18a-5p and miR-652-3p during early hospitalization was found to correlate with an increased risk of mortality within 180 days.^149^ Other circulating ncRNAs, such as lncRNAs, were also investigated for their potential as biomarkers in heart failure. Previous studies showed lncRNA UCA1 could predict a similar survival rate compared to BNP in patients with chronic heart failure.^152^ Similarly, quantitative analysis of lncRNAs in plasma revealed that NRON and MHRT have great potential as predictive biomarkers for heart failure.^153^

Other than biomarkers, ncRNAs are also attractive candidates for therapeutic targets in treating various human diseases.^154^ Some pioneering studies for cardiac regeneration with miRNAs have been carried out. Studies demonstrated that the miR-17-92 cluster plays a critical role in regulating cardiomyocyte proliferation in postnatal and adult hearts.^155^ A recent follow-up study explored the therapeutic potential of miR-19a/19b in protecting the heart in response to MI.^156^ In a MI mouse model, direct injection of miR-19a/19b mimics or AAV9-miR-19a/19b into infarcted hearts reduced scar formation, improved cardiac function, and promoted cardiomyocyte proliferation. Also note that transient overexpression of miR-19a/19b by injecting miR mimics has a long-term protective effect. Further investigation of the therapeutic effect of miR-19a/19b in a large animal model needs to be performed to demonstrate the therapeutic potential of this miR for MI in humans. Another miR, miR-199a, has also been shown to regulate cardiac regeneration. miR-199a promoted cardiomyocyte proliferation in both neonatal and adult rats.^157^ Excitingly, miR-199a also showed a therapeutic potential for MI in a large animal model. In an I/R injury pig model, overexpression of miR-199a in the myocardium using adenovirus-associated virus had a protective effect on the injured heart with better global cardiac function and regional/segmental contractility 28 days after injury.^158^ Further evidence demonstrated that morphological and functional improvements are associated with the role of miR-199a in promoting endogenous cardiomyocyte proliferation. However, it has been noticed that persistent and uncontrolled expression of miR-199a can cause sudden death due to arrhythmia. Therefore, several key factors, such as dosage, time window, and delivery approach, have to be carefully investigated before human trials can proceed.

A huge amount of effort has been spent on exploring targets and developing approaches for clinical applications in the diagnosis/treatment of cardiac diseases using proteins. Although great strides have been made, the clinical need has not yet been met. Numerous investigations of RNA regulatory networks, especially ncRNAs, will continue to provide new RNA targets with therapeutic potential. RNA targets have their own advantages as opposed to proteins, such as not relying on antibodies for their detection and their ease of synthesis and delivery. Therefore, in combination with protein targets, the discoveries of RNA regulatory networks will likely lead to a breakthrough in clinical applications for heart disease.

### Conclusion and Perspective

**Figure 1:**
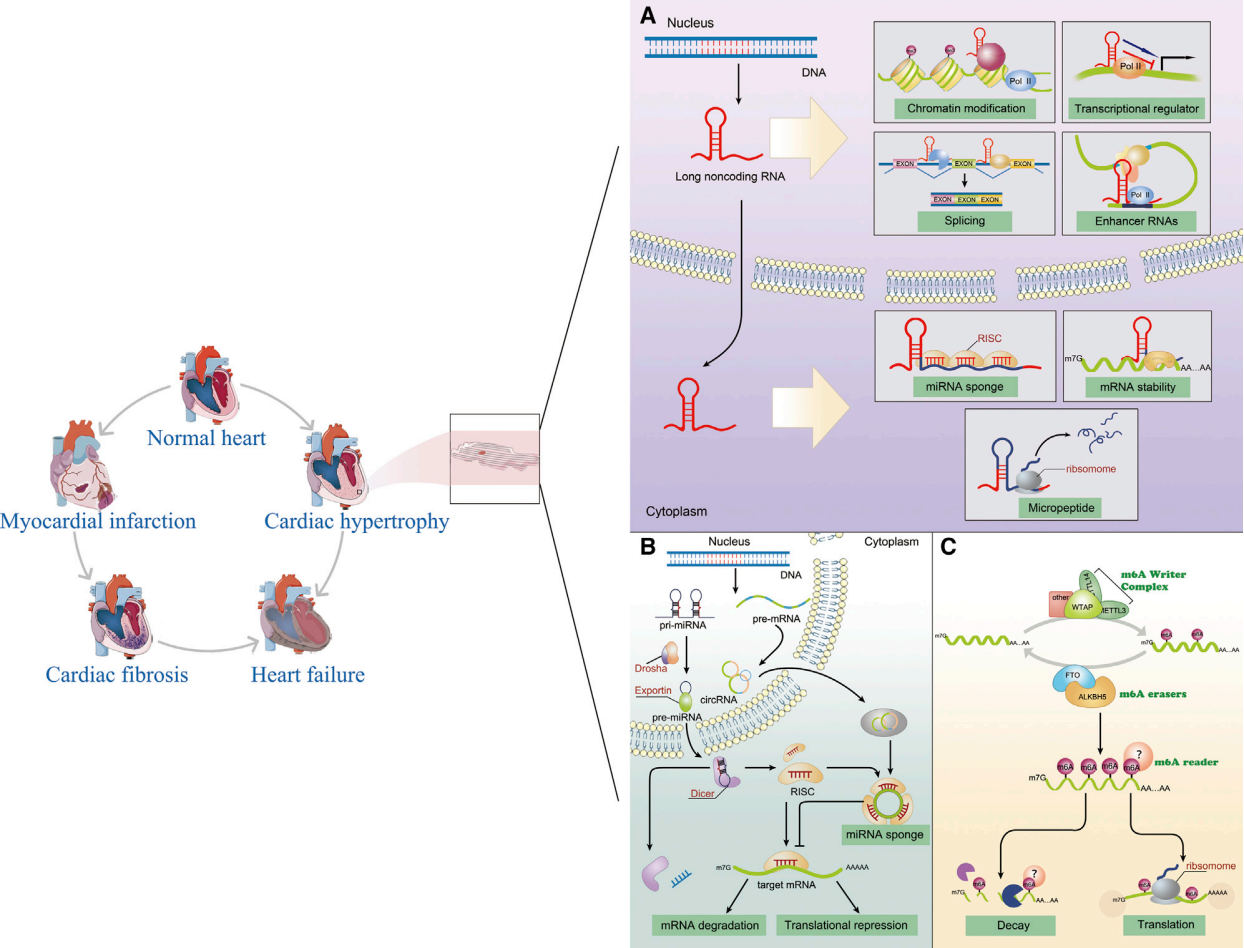
Molecular Mechanisms of Components of the RNA Regulatory Networks in the Heart (A) Molecular functions of lncRNAs in the heart. (B) Gene expression regulated by cardiac miRNAs and circRNAs. (C) m6A RNA modifications participate in the regulation of cardiac gene expression.

After annotation of the human genome, people surprisingly found that the amount of protein-coding genes and the length of coding sequences were comparable to many other vertebrates and even invertebrates, such as *C. elegans*. However, humans have more abundant non-coding DNA sequences than other lower species. Until the last two decades, ncRNAs started to be explored, and the known regulatory networks in cardiac pathology, which mainly consist of proteins, are likely to be just a “the tip of iceberg” phenomenon. In this review, we summarize the main discoveries in RNA regulatory networks in cardiac disease, which are just the beginning of exploring the “dark matter” of the human genome. Clearly, RNA molecules are one of an indispensable component of these networks. Further work will help us better understand the underlying molecular mechanisms of cardiac disease (Figure 1). Perhaps more importantly, this knowledge may provide a roadmap to defeat heart disease.

## Author Contributions

R.T., T.L., and Z.-P.H. prepared the manuscript. R.T. and T.L. wrote the main parts of the article and produced graphics. K.O.L. and Y.C. reviewed and edited the manuscript. Z.-P.H drafted the final version of the manuscript. All authors read and approved the final manuscript.

## Conflicts of Interest

The authors declare no competing interests.
